# Conceptualizing the impact of moral case deliberation: a multiple-case study in a health care institution for people with intellectual disabilities

**DOI:** 10.1186/s12910-022-00747-2

**Published:** 2022-02-05

**Authors:** J. C. de Snoo-Trimp, J. L. P. van Gurp, A. C. Molewijk

**Affiliations:** 1 Department of Ethics, Law and Humanities, Amsterdam UMC, VU University, De Boelelaan 1089a, 1081 HV Amsterdam, The Netherlands; 2 Department IQ Healthcare, Radboud University Medical Center, Nijmegen, The Netherlands; 3 Department of Ethics, Law and Humanities, Amsterdam UMC, VU University, Amsterdam, The Netherlands; 4 Center of Medical Ethics, Institute of Health and Society, University of Oslo, Oslo, Norway

**Keywords:** Moral case deliberation, Impact, Evaluation, Intellectual disabilities

## Abstract

**Background:**

As moral case deliberations (MCDs) have increasingly been implemented in health care institutions as a form of ethics support, it is relevant to know whether and how MCDs actually contribute to positive changes in care. Insight is needed on what actually happens in daily care practice following MCD sessions. This study aimed at investigating the impact of MCD and exploring how ‘impact of MCD’ should be conceptualized for future research.

**Methods:**

A multiple-case study was conducted in a care organization for people with intellectual disabilities and/or acquired brain injury, by observing MCD sessions as ‘cases’, followed by interviews with health care professionals concerning the follow-up to these cases, and a focus group with involved MCD facilitators. A conceptual scheme concerning the possible impact formed the basis for analysis: (1) individual moral awareness; (2) the actions of health care professionals; (3) collaboration among health care professionals; (4) the concrete situation of the client; (5) the client’s quality of care and life; (6) the organizational and policy level.

**Results:**

According to interviewees, their moral awareness and their collaboration, both among colleagues and with clients’ relatives, improved after MCD. Perceived impact on client situation, quality of care/life and the organizational level varied among interviewees or was difficult to define or link to MCD. Three aspects were added to the conceptual scheme concerning the impact of MCD: (a) preparations and expectations *prior* to the MCD session; (b) a translational step *between* the conclusions of the MCD session and practical events in the following period, and (c) collaboration with *clients’ relatives*. A negative impact of MCD was also found on misunderstandings among participants and disappointment about lack of follow-up.

**Conclusions:**

Concretizing and conceptualizing the ‘impact’ of MCD is complicated as many factors play a role either before or during the transition from MCD to practice. It is important to consider ‘impact’ in a broad sense and to relate it to the goals and context of the MCD in question. Future studies in this field should pay additional attention to the preparations, content and process involved in ethics support, including clients’ and relatives’ experiences.

**Supplementary Information:**

The online version contains supplementary material available at 10.1186/s12910-022-00747-2.

## Background

Various kinds of ethics support can be offered for health care professionals to deal with moral challenges [1]. In the Netherlands, ethics support is often provided in the form of moral case deliberation (MCD), which involves a facilitated collective dialogue concerning a moral problem taken from practice [2, 3]. As MCDs have increasingly been implemented in health care institutions [2], it is relevant to know whether and how MCDs actually contribute to positive changes in care practices. This information provides crucial insights into the extent to which MCD is actually a *supportive* service in practice. Furthermore, insight into the impact of MCD—like any ethics support service—might help guide the direction of the professionalization and implementation processes of MCD and to justify the use of MCD in a health care context [1, 4–7].

**Fig. 1:**
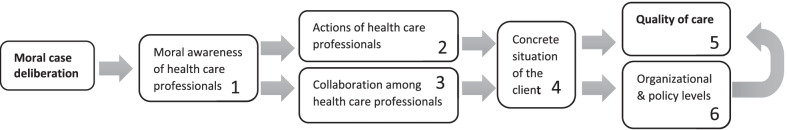
Preliminary scheme of the impact of moral case deliberation, according to the literature

We found several claims concerning the impact of (a series of) MCDs, on various levels, when exploratively looking for publications available before or in 2020, which are visualized in Fig. 1.

First, studies found that MCD participants reported being more aware of their own and others’ viewpoints and values with regard to moral cases [7–11]. Second, the impact on the actions of health care professionals is illustrated by one of the items of the Euro-MCD instrument, which aims to measure the experienced outcomes of MCD [10]: ‘[MCD] enables me and my co-workers to decide on concrete actions in order to manage the ethically difficult situations’. Third, cooperation among health care professionals might improve through MCD [10–13]. Weidema et al. [11] for instance showed that MCD was experienced by nurses as a factor that stimulated them ‘to ask for each other’s advice and expertise and to explicitly experience that they are not alone in their practice’. Fourth, impact on the concrete situation (without an explicit link to quality of care) of the client has been shown by Lillemoen & Pedersen [14], who indicated that MCD participants found new solutions resulting in concrete and significant changes in patient situations. Fifth, MCD has been viewed as positively but *indirectly* affecting quality of care according to participants in several studies [8, 11, 13–16]. In these studies, quality of care was not further defined, although Dauwerse and colleagues [16] mentioned that ‘improving quality of care for example means that the care actions (process), the organization and the care delivered (output) are qualitative high’. Finally, an impact on the organizational and policy levels has been suggested [10]. Although we do not want to claim that this overview is exhaustive, we believe that the six domains in which MCD could have an impact are sufficiently convincing to take as a starting point when studying the impact of MCD.

In all of these studies, the assumptions or claims were based solely on self-reports by health care professionals after having participated in MCD sessions, a fact which is also mentioned in the review on the impact of MCD by Haan et al. [7]. This limitation is not problematic when considering the first, second and third claims concerning moral awareness, actions and collaboration among health care professionals: to assess these factors, it is logical to (also) ask these questions of health care professionals *themselves*. However, assessing subjective experiences becomes less valuable in the context of investigating aspects operating on the meso level, separate from the personal experiences of the MCD participant, such as the client situation, quality of care, quality of life and impact on the organization (the fourth through sixth claims). The abovementioned studies that reported on changes in concrete care already acknowledged the lack of strong evidence concerning whether actual care was indeed changed and whether this change was caused by MCD, as they only assessed the ideas of participants on this subject, which do not always provide reliable information about the actual care provided [8, 11, 13, 14]. Haan and colleagues [7] also highlighted in their review the fact that ‘one should keep in mind that positive evaluations of participants do not necessarily imply that a group deliberation results in concrete changes in the way they treat their patient’. Claims concerning the impact of actual care should therefore be made with great caution. Even if there was a high level of impact on quality of care, it might not have been recognized or indicated by the individual interviewed MCD participant. In addition, it is important to conceptualize and clarify this ‘quality of care’ to identify any potential impact on it [4, 7]. As a result, it is, for the most part, unknown what *actually* happens in a concrete care setting after an MCD has taken place [7]. In their Cochrane Review, Schildmann et al. [6] could not arrive at clear conclusions regarding the effectiveness of ethical case interventions (such as MCD) on adult patient care, as they found only four randomized controlled trials with low certainty of evidence. The path from MCD to actual changes in care practices that improve quality of life and/or quality of care is still unknown. This is important to keep in mind when considering (especially steps 5 and 6 of) the conceptual scheme in Fig. 1.

Schildmann and colleagues [5, 6] studied the process elements of ethical case interventions such as MCD and characterized these interventions as ‘complex interventions’. They developed and promoted the use of ‘conceptual frameworks’ to explicitly visualize the ‘active ingredients’ of ethics support services such as MCD. Our scheme as presented in Fig. 1 can be seen as addition to this ‘conceptual framework’ as this scheme focuses on the events *beyond* the MCD instead of the active ingredients *within* the MCD [5]. As it is important for those implementing and executing ethics support services to know if and how the ethics support service actually leads to the assumed and intended changes in practice, we need a better theoretical and conceptual understanding of what actually happens in practice *after* the ethics support session. This requirement concerns both the practice of care for the individual patient and events at the policy or team level. This understanding will inform us when developing future research methods that fit this complex praxis of evaluating the impact of ethics support.

What method would then be suitable to adequately assess the actual changes in concrete care settings if assessments of the self-reported experiences of MCD participants, often health care professionals, are not completely suitable? We need to look at the care practices themselves after an MCD has taken place. Does care change as a consequence of moral considerations and new shared insights, and if so: how? For this purpose, case study research using observations of MCDs seems promising. This approach has been introduced primarily by Scandinavian researchers in the field of ethics support [17–19]. However, these researchers have focused on the content of MCD sessions and have not considered the context and follow-up. Additionally, views from care receivers and other outsider perspectives have not yet been studied, as acknowledged by Lillemoen and Pedersen [14], who wrote that ‘those who have received the service may consider it differently’. Only in a few studies were people who did not participate in the sessions involved in the interviews: managers [11, 12, 14], facilitators [14] and other key persons within the organization [9, 16].

Therefore, we want to study impact with a focus on the follow-up period after MCD has taken place: what happens in the weeks and months afterwards? We use the following definition of the ‘impact of MCD’: ‘*changes brought about by participating in MCD’* [7]. Because of the specifically normative nature of MCD (i.e., exploring how to define and substantiate ‘good care’), we consider change to be rather neutral and avoid making normative assumptions about reported changes [20, 21].

## Methods

### Aim

The primary aim of this study is to investigate changes in actual care practices as a consequence of MCD in the weeks and months following on care professionals, clients, care practices and perceived quality of life and quality of care, in a care setting for people with intellectual disabilities or acquired brain injury. Considering the concept of quality of care mentioned in the Introduction, we would also like to focus on quality of life in this context, as this concept is more apparent and relevant to the context of intellectual disabilities. Robert Schalock [22] defines quality of life by describing eight domains: emotional wellbeing, interpersonal relations, material wellbeing, personal development, physical wellbeing, self-determination, social inclusion and rights. It is wellknown that health care professionals in this care setting face moral challenges throughout their daily work [23]. As conceptualizing and developing methods of assessing this impact is challenging but necessary, as outlined in the Introduction [4, 7], our second aim is to explore how to think about and empirically research the impact of MCD.

### Design

We applied the multiple-case study approach described by Robert Yin [24]. This approach is ‘particularly well-suited for extensive and in-depth descriptions of complex social phenomena’ [25] because the inquiry focuses on both phenomenon and context and can include various research methods [24]. MCD sessions can be understood as ‘complex social phenomena’ since MCD involves diverse stakeholders interacting with one another on various levels; they involve specific skills and knowledge on the part of those facilitating and attending the MCD session; and it is implemented, performed and evaluated in a variety of ways, indicating various potentially relevant ingredients for impact, as shown in Fig. 1, and described by Schildmann and colleagues [5]. We explored all these levels in the context of a specific MCD session (the case), over time, resulting in rich data to investigate the follow-up to and actual impact of that MCD session. Furthermore, this approach allows for so-called ‘analytical generalization’ [24]: findings can support or add to existing theories about social phenomena. Therefore, to conceptualize impact according to our second aim, we took the conceptual scheme of impact described earlier into account while collecting and analyzing data: to what extent do observed MCD sessions and related follow-up activities confirm or contradict existing descriptions of impact of MCD in literature? As such, a multiple-case study can ultimately provide a comprehensive overview of the impact of MCD sessions on actual care practices.

### Setting

The study was conducted in a large Dutch health care organization for people with intellectual disabilities and/or acquired brain injury. This organization provides a wide range of care facilities, including ambulatory services, assisted living facilities, centres for daytime activities and both daily and overnight childcare. Care is provided by teams of health care professionals from various disciplines, such as direct support staff, therapists, behavioural experts, psychologists, physicians, social workers and managers. MCDs are frequently organized throughout the organization, sometimes including clients or their relatives as participants. MCDs are facilitated by health care professionals from the organization who have been trained in the dilemma method [3, 26]. The dilemma method consists of 10 steps through which the group is led through the dialogue, including determining the aim of the session, presenting and clarifying the case, analyzing the case from relevant perspectives, brainstorming about alternatives, sharing and considering individual viewpoints and eventually coming to conclusions, after which the session is ended by evaluating both the content and the process of the MCD session [3].

### Data collection

Convenient sampling was used to collect data, as MCDs that were planned within the organization during the study period (January 2018 to April 2019) were included for observation by a researcher (JS). After the observation, the initial findings concerning the content, process and participants were discussed by the research team (JS, JG and AM) to decide whether the MCD would be selected as a case for follow-up. The inclusion criteria were (1) multiple stakeholders being present during the MCD, preferably including clients and their relatives; (2) a clear moral dilemma concerning a concrete patient situation being at the core of the dialogue; (3) openness by all MCD participants to talk about the case during the session; and (4) the agreement of all MCD participants to be observed by the researcher during the MCD session. The exclusion criteria were (1) MCDs remaining incomplete due to time restraints; (2) MCDs in which multiple and diverging dilemmas related to different patients were discussed; and (3) no possibility of following up on the case due to external factors such as the departure of the client. If a case was selected, interviews were planned by JS with as many as possible participants of the session and, if possible, also with other relevant stakeholders such as client and their relatives. These individuals were invited with a formal invitation letter via e-mail explaining the study details. Furthermore, the facilitator’s report of the session was collected for comparison with the observer’s notes.

An observation guide was constructed by JS and JG to list relevant aspects and guide observational notes (Additional file 1: Appendix I). This guide contained elements from the Descriptive Question Matrix by Spradley [27] and was inspired by the example of Hanson [28]. The guide contained the following parts: (1) global overview of the session; (2) relevant aspects prior to the session; (3) the MCD itself, including the following aspects: (a) room and objects, b) participants, (c) the case, (d) considerations, (e) conclusions and (f) the role of the facilitator; (4) relevant aspects directly after the session.

The interviews were semistructured, lasted between 12 and 50 min and were held by JS at a place selected by the respondent. The interviews started with a reflection on the MCD session, including the background, case, dilemma, participation, conclusion and respondent’s feelings during and following the session. Second, the respondent was asked about the period since the MCD session, focusing on the situation of the client and whether this situation had changed, the way of caring for the client (personally and as a team) and any follow-up to conclusions made in the MCD session. The interview concluded with questions about experiences with MCD in general and the respondent’s ideas concerning the value of MCD and possible conditions for or barriers to greater impact. The interview guide is shown in Additional file 2: Appendix II. The interviews were audio-recorded and transcribed by JS and a student assistant who signed a confidentiality agreement for this purpose.

The facilitators of the observed MCD sessions were invited by JS to a focus group meeting (lasting two hours) facilitated by JG to discuss the preliminary findings from the observations and interviews and to gain insight into their experiences in facilitating MCD and their ideas concerning the impact of MCD within their organization. As such, the focus group served as a member check: to assess the credibility of the findings and their connection to experiences in practice and to add depth to the overall analysis. The programme for the focus group meeting is presented in Additional file 3: Appendix III. The meeting was audio-recorded, and a summary was made by JS by re-listening the session afterwards (no word-for-word transcription was made).

### Ethical considerations

Participants in the MCDs to be observed were informed about the presence of a passive observer beforehand, with it being explicitly stated that they were free to express their possible objection to the facilitator. At the start of the MCD, the presence of the observer was explained again to ensure that everyone agreed with the observer’s presence. For the interviews, invitation letters were sent in which information was provided about the aim and background of the study. In these letters, it was also explained that participation was voluntary, that answers would be treated confidentially and that all personal aspects would be deleted from the stored data. The invitation letter for clients was adjusted to use easier language and made more accessible via pictograms in accordance with Dutch guidelines for research conducted with people with a mental disability [29]. Before the start of each interview and the focus group meeting, it was mentioned that the researchers had no personal affiliations with the organization or a personal interest in the success of MCD, and written informed consent was obtained. After transcription and summarizing, the audiotapes of the interviews and the focus group meeting were deleted. Interview transcripts and the focus group summary did not contain any personally identifiable information and were saved in secure computer files for a maximum of 5 years. The study protocol was submitted to the Institutional Review Board (CMO) of Radboudumc, who declared no objection to the conduct of this study and also declared that no further ethics approval was needed according to national regulations (Ref. no. 2018-4259).

### Data analysis

The starting point for analysis was the visualization of impact presented earlier, based on previous literature (Fig. 1). We considered this tentative framework to be preliminary and used it (a) to sensitize the researcher to relevant nuances in the data and (b) as ‘a source for making comparisons’ [30]. Focusing on conceptualizing the impact of moral case deliberation, this tentative framework provided a starting point and direction for both data collection and analysis while remaining open to new conceptual insights [30]. This conceptualization of the impact of MCD is most credible when it connects to the existing literature and knowledge as well as to the empirical facts [31]. The analysis was conducted over several steps (see Box 1): after familiarization with the interview transcripts, codes and categories were revealed by open and axial coding, which were compared with those from other interviews and the observational notes for that MCD session. The result of this within-case analysis was also compared to the tentative framework (Fig. 1), following the method of ‘pattern matching’ [24], to assess whether and how we could further refine the theory and fill in logical gaps within a single case [30]. Then, we performed a cross-case analysis: we compared the overviews of all MCDs and integrated them into an overall overview of (hypothesized and new) elements for (attaining) impact. This preliminary overall overview was presented to and discussed with facilitators in the focus group, after which we first created a separate conceptual scheme solely on the basis of their input, which was subsequently integrated in the preliminary overview. Finally, we adjusted the conceptual schema, which is described and reflected on in the Discussion section. During the whole process of analysis, we wrote memos concerning the decisions made in the integration process. See also Box 1 for the roles of the research team members in this process.

**Box 1:** Overview of steps during data analysis

	Researcher(s)
**Within case**	
1. Familiarization with interview transcripts and observational notes through reading and summary	JS
2. Open and axial coding of interview and observations, resulting in codes and categories, comparison of codes and categories between interviews and observations from same MCD	Pairs of authors
3. Developing a visual overview for each case by inserting categories and patterns into a conceptual scheme of impact (Fig. 1), as confirming or additional element	All
**Cross cases**	
4. Cross-case analysis: comparing visual overviews (categories and patterns) of multiple cases (MCDs) to develop a preliminary conceptual scheme representing all findings	Initiated by JS, discussed in pairs per MCD
5. Member check with focus group members by presenting the preliminary overall scheme	All
6. Developing a visual overview of focus group input into the (separate) conceptual scheme	JS and JG
7. Integrating focus group findings into the conceptual scheme and finalizing conceptual scheme of impact	Initiated by JS, discussed by all authors

During all steps: writing memos concerning decisions made

## Results

In total, eight MCDs were included in the study. During the study period, approximately 80 MCDs took place throughout the whole organization. Within the organization, the selected sessions represented a variety of settings for MCDs as well as participant professions and the presence of clients/clients’ relatives. Six of eight MCDs were observed, and two were added on the basis of the facilitators’ report. One of these two MCDs took place before the start of the study and was included to assess what participants still remembered or noticed about the MCD and, as such, to gain insight into the long-term impact. The other MCD was not observed for practical reasons but was deemed relevant since clients’ relatives took part in the session, as only one other (observed) session included a client relative during the study period to our knowledge. The facilitators of these sessions gave their consent to use their reports concerning these nonobserved MCDs. These reports did not include any identifiable information related to participants or clients and were publicly available within the organization. In the Netherlands, ethics support activities and reports are not part of client files and do not require the consent of clients and/or their relatives. Relevant details, including setting and participants, are presented in Table 1. Due to privacy and confidentiality concerns, we do not distinguish among various professions when presenting the viewpoints of health care professionals in subsequent parts.

**Table 1:** Characteristics of included moral case deliberations (MCDs) and interview respondents

Setting	MCDs	Participants per MCD (N)^1^	Interviewed participants (N)
Day care and activity centers	3	5/7/8, including direct support staff, managers and behavioural experts	11
Ambulatory care services	2	6/5, including direct support staff, therapists, managers and clients’ relatives	4
Assisted living homes	2	6/9, including direct support staff, therapists, managers and clients’ relatives	6
Childcare centres	1	7, including direct support staff and behavioural experts	1

Total: 8 MCDs and 20 interviews with 22 health care professionals who participated in MCD

^1^There were no participants who attended more than one included MCD session

### Observations of the MCD sessions

The MCDs were focused on various dilemmas, which in some cases had already existed for several years. The conclusions varied as well, from less to more concrete plans for practice (see Table 2 for an overview).

**Table 2:** Overview of the MCD sessions

MCD #	Moral dilemma *as defined by the group in the first part of the session (3)*	Conclusion of MCD session
1	Should I inform the Inspectorate about our worries about the home situation of our client or is the home situation beyond the limits of our care?	Intention to be more open to connection with client’s relatives; no plans made for practice
2	Should I keep the client in the group activity room or allow the client to move around in the building when the client is restless?	Intention to discuss practical actions in subsequent meeting
3	May I share information from a confidential talk with a client with my team or should I respect the client’s wish not to share it?	New consensus on existing statement
4*^	May we leave the client alone when taking care of clients in another building or should we take the client with us?	New consensus on existing statement and making plans for practical consequences
5*	Are we allowed to stop home care to protect care workers´ health and safety, or is it our duty to continue home care for this client?	Making plans for concrete actions
6^	Should we go along with the wishes of the client’s relatives or should we set a limit, based on our professional expertise?	Making plans for concrete actions
7	Should we stop home care for this care-refusing client, or should we continue and intensify care with out-of-home placement?	Making plans for concrete actions
8	May I apply a freedom-restrictive tool to this child when parents’ wish it to be applied, or may I refrain from using it, as I feel doubtful about its effectiveness?	Open ended, no plans made for practice

*Clients’ relatives present

^Not observed, included on basis of facilitators’ report

### In the period after MCD: Characteristics of interviewees and focus group participants

Interviews were held with participants of the MCD at their workplace 2–7 months after 7 of 8 MCDs took place. For the MCD that took place prior to our study, participants were interviewed 14–16 months after the MCD had taken place. Despite our initial plans, interviews were only conducted with health care professionals. In two MCDs (#3 and #4 in Table 2), client relatives participated as well, but the relationship between those relatives and the health care professionals was too problematic to send an interview invitation (in one case), or the relatives refused the interview invitation because of strong emotions about the situation (in the other case).

Participants in the focus group included five of seven invited facilitators, who had a leading or assisting role in facilitating one or more observed MCDs. These facilitators worked as direct caretakers, therapists or pastoral workers in the organization. During the session, the interview findings were presented according to the conceptual scheme, supplemented by newly found elements, and facilitators were asked for their comments, adjustments and additions regarding the impact of MCD.

In the following sections, we describe our findings concerning the impact of the included MCDs, integrating input from both the interviews and the focus group. Our findings will be structured according to the conceptual scheme presented earlier (Fig. 1), supplemented by new elements. First, we describe several determinants that are crucial to achieve impact. Then, we describe several domains in which impact was perceived. Finally, we also describe two forms of negative impact that we encountered during data collection.

#### Crucial aspects prior to the MCD sessions that influence the possibility for impact

Especially during the observations of the MCD sessions, we learned that several events that had occurred prior to or during the MCD sessions played a crucial role in realizing possible impact afterwards, which were not yet included in the conceptual scheme (Fig. 1). These aspects were confirmed in the interviews and the focus group meeting. First, the attitude of MCD participants before entering the MCD influenced the process and outcome of the session. In general, we learned about three attitudes before entering the MCD: open, sceptical or prejudiced. Facilitators stressed the importance of clarifying expectations concerning and the perceived goals of the session at the start, as these factors might differ among participants and help to articulate a clear shared focus for the MCD session itself. Most participants had no prior experience with participation in MCD and entered sessions with an open and curious attitude, although some were sceptical about the potential of MCD to ‘solve’ the moral issue in question. Both attitudes still seemed to allow facilitators and participants to postpone personal judgment and openly investigate alternatives. For a few interviewed MCD participants, the endpoint was already clear, and they saw the MCD as time to assess their own judgment and actions. For that reason, they seemed more reluctant to look for alternatives, and they might have felt judged by the other participants when being questioned about their actions during the MCD session.

[The MCD] is a kind of assessment, assessing what I had done. Is that right according to all of us? […] The discussion has taken place, the moment of reflection has passed. I have achieved that goal. […] And in the end, it has been determined, I thought the conclusion was, that it was done well.

A safe atmosphere to talk and an experience of felt urgency among participants to do something about the issue were also suggested to be crucial aspects for realizing impact by facilitators, as a means of openly exploring new options and motivating further action. Facilitators mentioned in the focus group meeting that they had experienced that participants often said they were relieved or motivated to move on with the case at the end of the MCD session. Facilitators further mentioned two challenges in facilitating impactful sessions: finding a clear and to-the-point dilemma to address the core moral question and subsequently an in-depth dialogue and reserving sufficient time to create practical plans.

#### Impact on moral awareness

In most MCDs, participants felt positively at the end of the session. Several interviewees described that MCD affected their way of thinking about the situation by teaching them to look at the situation from a distance and allowing them to discover new insights into situations. Because of this change, they reported looking differently at clients, clients’ relatives and their own actions. They also said that the MCD helped them clarify the situation and become empowered to articulate personal viewpoints or team decisions to clients and their relatives.

Furthermore, the MCD provided room to express frustrations, and through such expression, participants felt relieved and acknowledged. Some interviewees said that because of the space in which it was permitted to share emotions and experience support during the MCD, they felt more self-confident in dealing with the situation afterwards and were more inclined to report their frustrations earlier in the future.

We have shed tears there but it [the MCD] helped, it gave us such a relief. And from then, we were really able to make the whole situation better. And it worked from day 1. It has strengthened us so much! And it has already been more than a year since then now, I realize, and it is still noticeable.

#### Impact on collaboration among health care professionals

Already during the MCD, participants mentioned the added value of the MCD for overall collaboration among them by producing a better understanding of and more respect for one another’s viewpoints. Interviewees also described that, after the MCD, they and their colleagues talked more often and more openly about the situation. This change also led to more trust in one another and feelings of being supported by colleagues *afterwards,* for instance, by feeling less inclined to presuppose that colleagues have negative opinions of one’s own way of acting.

…I indeed assumed that my colleagues would find us, so to say, ‘annoying’. […] But indeed, that was not the case! […] … What I still clearly remember is that colleagues from other groups supported us very much and that they had a lot of understanding. So… that was actually the only thing that has stayed with me, about which I thought, okay, we are not alone in this. We are seen, in a positive way.

Furthermore, the collaboration itself changed after MCD, according to some, for instance, by using ‘techniques’ from the MCD in regular team meetings, such as allowing everyone an explicit moment to give his or her opinion about the issue. One interviewee said that team discussions also improved and that MCD thereby helped to formulate a clear and team-based approach to the client.

…the moral case deliberation [is] where you look each other in the eyes and say ‘yes I think this is acceptable’ […] and that there then is a person next to you who says ‘no I don’t think this is acceptable’. But in the end, you all get round the table and say, okay, but we do agree now that we should go this way here.

#### Impact on collaboration with clients’ relatives

A form of impact beyond our hypotheses (Fig. 1) concerned collaboration with clients’ relatives. Some interviewees mentioned that *during* the MCD sessions in which relatives of the client participated, a better understanding of each other’s situation and viewpoints was attained.

…you are with people who all look differently to that person, and who all envisage the same thing, but the ideas are different for everyone. A mother looks completely different to her child than that we look, and a manager looks in again another way. […] And in the end, the facilitator was so well able to bring that together. I really found that so super. […] I think that, considering the insights into one another, it has been a tremendously good conversation.

In MCDs in which the moral problem had to do with a relationship with a client’s relative (#1, #6, #7 and #8, Table 2), interviewees reported that the MCD led to a different approach to relatives, for instance, by allowing the interviewee to feel more competent and better able to explain the care plans.

…[the MCD] did help to clearly articulate things to parents, so that we can better explain why we can do or cannot do certain things.

Some also said that they were more respectful of clients’ relatives, as they now better understood why the family acted the way they did. Some interviewees also thought that relatives (who did not participate in the MCD) were also acting in a different way after the MCD, by becoming more open and understanding and expressing more trust and satisfaction towards interviewees.

Health care professionals often said that clients’ relatives were consciously *not* informed about the MCD because they feared that an MCD would worsen the—already problematic—relationship with relatives or because they used the MCD as a way of producing a team-based consensus before involving the relatives of the clients.

#### Impact on the actions of health care professionals

For four MCDs (#1, #2, #6 and #7, Table 2), health care professionals described that their way of acting changed in the period after the sessions, particularly in terms of their approach to clients’ relatives. Some interviewees could not answer the question of whether the MCD had affected their actions, as they were still working on certain actions that were initiated during or due to the MCD (in #5, #7 and #8). Some interviewees felt a need to first concretize the conclusions made during the MCDs into actual plans for practice. They said that the MCD gave an initial boost to start these actions, as they all felt the same urgency to deal with the issue in that context.

…I think that, because it has been going on for so long, we are continuously discussing it with each other. But at the same time, it was going to smolder a bit, like well, it is what it is and oh we should do something with it, some day. And I do think that the moral case deliberation was a boost, like oh we should really do something with it, quick, and that it did contribute to a number of actions that have been done now.

#### Impact on concrete situation of client

Worries about the wellbeing of the client (at home or at the care facility) were the starting point for several MCDs (#1, #2, #4, #5 and #7). In some MCDs (#3, #6 and #8), the focus was on team communication or contact with clients’ relatives, focuses which were indirectly related to the client as well. In the period after the sessions, most health care professionals indicated that they still worried about the client’s situation. Some said that they had not expected that the situation of the client would improve due to the MCD, as improvement would require changes on levels beyond their grasp.

Interviewees had various opinions concerning whether the situation had improved in the period after the MCD sessions, sometimes even when discussing the same MCD session. In the context of one MCD (#7), one interviewee said that the client situation would not have been different if the MCD had not taken place, but another professional felt that the MCD gave a new and helpful stimulus for taking concrete steps to improve the situation.

Facilitators mentioned during the focus group meeting that they did not know whether the conclusions of the MCDs were followed or whether the situation of the client had changed. Some facilitators said that they would like to keep in contact with the team in the subsequent period, but others said that follow-up to a case is the responsibility of the team. The facilitator should stimulate the team as much as possible *during* the MCD to take this responsibility.

#### Impact on quality of care and quality of life

Interviewees did not explicitly or spontaneously mention any impact on the quality of care for or the quality of life of their clients. Some interviewees mentioned that the MCD led to changes such as better contact with the relatives of the client, resulting in more information about how to care for the client and a more stable situation. In other cases, interviewees said that MCD helped them to focus on the client again instead of on problematic contact with clients’ relatives. Interviewees also mentioned that the MCD session led to more freedom for the client. All of these comments can be interpreted as examples of impact on quality of care, but only indirectly.

Two MCD sessions (#1 and #7) resulted in a decision *to refrain from* drastic measures (such as out-of-home placement). Some interviewees said that, according to them, these drastic measures were unrealistic in the first place (i.e., even before the MCD session). This specific interpretation by some interviewees therefore makes it difficult to directly link this decision to the ‘impact of MCD on quality of care’. It seems that MCD at least provided space for an in-depth and critical inquiry into this apparently unrealistic option, leading to a more justified confirmation of the preassumed decision. Indeed, according to some interviewees, the MCD helped them to see that the measure would truly be traumatizing for the client and that the decision to withhold this action was best for the client, at least in the shortterm.

When explicitly asked about the impact of the MCD on quality of life, interviewees were hesitant because they were doubtful concerning what quality of life would look like in a particular client’s situation. Some interviewees mentioned that they gained new insights about quality of life for the client during the MCD session, which might implicitly affect quality of life as well.

If you are talking about quality of life, and you look at the client, what is then very close and important to him? Well, his family. Yes. And not that he gets a clean diaper in a timely manner, or that his chair fits his disability exactly. That is not in his experience, though it is for his sake, but not for his senses.

In the focus group meeting, facilitators recognized this struggle concerning how to define quality of life and who should define it, but they felt that since MCD was introduced in the institution, attention to the perspective and values of clients had increased. Hence, they were convinced of the contribution of MCD to the tasks of locating the client closer to the centre of care and focusing on their quality of life. They suggested that the meaning of quality of life or quality of care in a particular situation is sometimes exactly the question of the MCD, leading to a solution on which everyone agrees, *‘where we all say: this is quality of care’.*

#### Impact on the organizational and policy level

The hypothesized impact on the organization and policy level did not appear in the responses of the interviewees. However, some interviewed managers mentioned that they planned to use (parts of) MCD more frequently to stimulate moral reflection among their team in a more structured way. Facilitators mentioned in the focus group meeting that they saw MCD as a welcome opportunity in the organization for saving time and space to discuss long-lasting problems.

#### Negative impact: misinterpretations and lack of follow-up

In one of the observed MCDs, a client relative was present. This relative was also a client himself but took part as the representative of another client (his partner). During the MCD, it was observed that his participation was rather limited; he did not seem to understand the dialogue and was not involved in the decision-making process concerning the actions to take, despite explicit attempts by both facilitator and participating caretakers to treat him as an equal participant. According to one of the interviewed caretakers, this MCD session was harmful to this client because it worsened his (already delicate) relationship and led to persistent misinterpretations and stress in both this client and the client that he represented. In the period after the MCD, caretakers had to put a great deal of effort into resolving this misunderstanding and rebuilding trust.

[Client] took it so literally. For example, there was something said like ‘let’s call a cleaning crew to come!’. Well, you must know, he still talks about this. He takes that deadly seriously, he didn’t see that as a joke. […] It can make him upset for a year. […] And that has led to so many tensions, we had to put many efforts to set things right a bit.

Another form of negative impact was the disappointment expressed by some interviewees about the lack of action in the period following the MCD session. They said that they were enthusiastic about the MCD because all involved parties felt the urgency to do something about the stressful situation, and that the MCD gave them a hopeful feeling that things would change soon, but they maintained that this change did not happen, unfortunately. For some, this anticlimax gave them ambiguous feelings concerning the impact of the MCD. These interviewees missed an explicit follow-up step to carry out plans in practice. There was no consensus among facilitators in the focus group meeting about their role or responsibility in monitoring or encouraging follow-up.

Really, the conversation wasn’t a waste of time. Absolutely not. It has brought us much more good results than I had ever expected, already when just considering the way of understanding each other. So that is really nice. But I think that there should be done more in the implementation of it. […] Be transparent and open about the follow-up and if you do so, make sure that the flow keeps going. And now it is actually like: it is what it is. And the conclusion actually was that it was not an optimal situation [for the client]. […] But why has nothing yet been done then?

## Discussion

The aim of this paper was to study the impact of MCD by focusing on the period *after* single MCD sessions and to explore how the ‘impact’ of MCD should be conceptualized. Our findings first show that health care professionals and facilitators explicitly reported an impact on their individual moral awareness and their collaboration with their colleagues and clients’ relatives. For some individuals, this impact also changed their concrete actions. As we described in the Introduction, a self-reported impact on moral awareness, collaboration and concrete actions has also been found in literature [4, 7–14].

### Adjusting the conceptual scheme

**Fig. 2:**
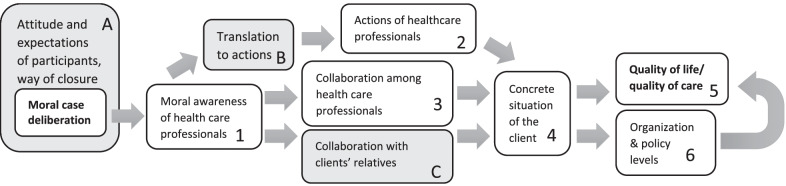
Adjusted scheme of the impact of moral case deliberation, according to the literature

Our findings indicated that the conceptual framework had to be adjusted, as presented in Fig. 2. Three aspects have been added: (A) relevant aspects prior to and during the MCD session; (B) a translational step between the session and actual practice; and (C) collaboration with clients’ relatives.

First, several aspects *prior to and during* the MCD determine the potential for impact: practical preparations, participants’ attitudes and expectations (regarding the MCD and the possibility to change practice), the atmosphere and content of the session and the way of closing the session and concretizing plans for practice. As such, this finding does not concern impact itself but can be seen as a ‘confounder’ that to a great extent can predict or clarify the kind of impact that is or will be realized. We observed in one MCD that due to careless preparation, the trust of and relationship with the participating client was harmed during the MCD, despite others’ efforts to involve him. This harm potentially damages impact *beyond* the MCD session itself. We learned here that, in preparing the MCD, assessing people's ability to join the (rather cognitive) discussion and their capacity to look at the case from a critical distance is crucial to avoid these negative consequences for these vulnerable people. This preparation could, for instance, include a plan concerning how to talk *with* these clients at their level of understanding instead of *about* them.

In a way, it is not surprising that impact can be facilitated or disturbed by the content and process of the event (the MCD) since that is why it is called ‘impact’ and why we initiated this research in the first place. However, these claims do not yet have a strong empirical basis. Additionally, no consensus or clear quality criteria exist concerning what the process and content of an MCD session *should* be [32, 33]. The reason for encountering this new aspect is partly due to the observational nature of our research design, which has enabled us to literally ‘see’ these relevant aspects as inherent to the process of the MCD (such as the atmosphere and attitude of its participants). As such, our findings shed light on a new research area: what is happening within the ‘black box’ of MCD [4, 5]. Further research is therefore recommended to investigate both the preparation as well as the process and content of the MCD session itself.

Second, the conclusion or plans for action of the MCD did not always turn out to be implemented in practice. A translational step is needed between (the conclusion of) the MCD and practical plans for change. This step was not always taken because of a lack of time in several MCDs. Another possible explanation for the lack of (and need for) this translational step is that, in the MCD, participants are challenged to take a step back to look at the case from a critical distance, postpone their intuitive decisions and consider out-of-the box alternatives to gain new insights. This ‘distant view’ might, however, complicate a concrete plan for practice, or at least, it needs a ‘landing ground’ to connect distant insights to practical actions. It is important to pay attention to this translational step and the practical follow-up, as we also found a potential ‘negative’ impact here in terms of a perceived lack of action after valued positively valued MCD. Here, this anticlimax was felt by those who were dependent on others to take action. The perceived lack of follow-up made them feel even more alone after the experience of being acknowledged during the MCD. This finding underlines the importance of not only evaluating the experiences of MCD, as these might be (excessively) positive when compared to actual consequences in practice. It might help to clarify and perhaps extend the task of the facilitator in guiding MCD participants to make decisions concerning concrete actions (e.g., who is doing what and when?) in order to prevent feelings of disappointment. In addition, this finding also shows that it is important to know whether people actually felt any urgency to undertake actions discussed during MCDs, to determine whether a lack of effect or follow-up was indeed contrary to their expectations and hence might be seen as a negative impact.

We need to note that defining a ‘negative’ impact is not easy. This difficulty is not only because it is not certain whether unpreferred consequences are directly due to the MCD but also because what ‘negative’ and ‘positive’ mean might differ among contexts and requires thorough justification [20]. Therefore, we want to avoid providing a statement concerning what ‘negative’ impact entails, rather, we only want to show what our data suggest, in close proximity to the perceptions of the interviewed health care professionals.

Third, a clear new form of impact appeared in our data: collaboration with clients’ relatives. Although the literature has not strongly indicated this impact, it is not surprising when considering the setting of our study. Like any long-term care facility, contact and collaboration with relatives is continuously crucial to provide the best possible care for the client, especially when clients are themselves less capable of decision-making. This long-lasting and enduring collaboration is vulnerable to misunderstandings or distrust on the part of both parties. Hence, moral dilemmas might rise and even last for (many) years. Our data show that, to some extent, MCD helped health care professionals clarify these long-lasting dilemmas, better understand the position of clients’ relatives and change their attitudes or approaches towards clients’ relatives. At the same time, it is important not to overestimate the role of MCD in this context, since it is a single event in a process that includes many moments of contact between health care professionals and clients’ relatives. The exact impact on collaboration with clients’ relatives might even be doubtful when considering that MCD is sometimes used by health care professionals without informing clients’ relatives (out of fear of conflict) and/or with the primary aim of finding a team-based consensus.

### Measuring impact on quality of care and quality of life

In our study, the concrete impact on the wellbeing (in terms of quality of life and quality of care) of clients appeared to be complicated. As mentioned, we did not find concrete evidence concerning the impact of MCD on quality of life and quality of care. In addition, we are well aware that our study does not completely do justice to these broad and different concepts of quality of life and quality of care. Especially the concept ‘quality of life’ related to the impact of MCD seems rather invisible in literature yet. These two concepts should therefore receive in-depth exploration in future studies concerning impact of clinical ethics support services like MCD. The interviewed health care professionals and facilitators found it hard to point to any direct impact in this context as they often doubted whether the client situation had changed or could change and if so, whether that change was a result of the MCD—possibly due to the long-term care setting as mentioned before. Therefore, we purposely distinguished (in the conceptual scheme and the findings) between impact on the client situation and impact on quality of care and quality of life. The main difficulty lies in the concepts of quality of care and quality of life itself: how to define these concepts and according to which perspective? The question about what quality of life or quality of care entails is often a reason for healthcare professionals to initiate an MCD. Especially considering our study context of long-term care for people who cannot always easily express their own wishes, it is important to continuously discuss the question of what quality of life means and who is able to define it. Even given these discussions, consensus will not always be found. It can therefore be challenging to measure the impact of MCD with standardized questionnaires based on predefined concepts or descriptions of quality of care and quality of life, as these concepts are redefined and continuously finetuned during the MCD session. Hence, preset measurement instruments for quality of life might not fit a) the inherent normative uncertainty in MCD regarding what quality of life means and b) the context-dependent conclusion of an MCD session. Recently, Haltaufderheide and colleagues [21] also indicated this difficulty when evaluating ethical case consultations: *‘ethical case consultation] services are usually complex and dynamic entities evolving over time and influenced by many different factors. Their normative standard rarely exists as a detailed ethical theory, but is often an implicit part of operational practices’.*

Furthermore, it is important to note that, when conceptualizing ‘impact’, the intention of participants might be to think about ‘changes’ and ‘actions’, while it might well be the case that MCD leads to no change at all, or to the confirmation of an already existing decision (as our data suggests), or even a decision *not* to perform a certain action (as our data also suggests). In addition, it is important to consider participants’ expectations of and wishes for achieving change in practice and to account for the goals they envision for the particular MCD session. For example, MCD can be deployed with the aim of sharing reflections. The concept of the impact of MCD does not therefore imply that MCD *should* always lead to change. Reflecting on the definition of impact that we presented earlier (i.e., ‘changes brought about by participating in MCD’), we need to add the claim that these changes should at least be in line with both the goals and decisions made during the MCD session: ‘Either change, or rejection of change (at least partly) brought about through participating in the MCD session(s)’.

### Impact on the organizational level

A possible impact on the organizational level was rarely and not explicitly mentioned by our interviewees. In our conceptual scheme (Fig. 2), the impact on the organizational level is positioned at a similar distance from the MCD box as the quality of care/quality of life box. This distance indicates that here too, a direct link between MCD and impact cannot be easily established. However, it is important to note that most of our interviewees were not involved in organizational matters, so an impact on this level might not have been noted by them. But the reverse situation is also the case: an impact at the organizational level might require an extra step to apply insights from practice to the institutional level. A strong and structural implementation of MCD is usually needed before this kind of impact can be detected [34]. Nevertheless, we believe that MCD can and even should impact the organizational level of making, adjusting and monitoring institutional policies.

### Methodological considerations

The multiple-case study design was a suitable method for studying the concept of the impact of MCD and for discovering new conceptual areas by focusing on the follow-up period after single MCD sessions in an actual care setting. Our observations of MCDs were of great value gaining insight into relevant ‘active components’, which have not been given much attention in MCD research to date [5, 6]. At the same time, our design was time-consuming and did not, in this project, allow for the inclusion of many MCDs or a series of consecutive MCD sessions or for comparison of many participants’ perceptions. To obtain a broader picture of the impact of MCD, based on more participants and more MCD sessions, qualitative methods can be complemented by quantitative methods that fit within this context-sensitive praxis. Recently, a short questionnaire has been presented to assess the impact (outcomes) of MCD, which contains 15 items related to moral competence, moral teamwork and moral action: the Euro-MCD 2.0 [4]. This questionnaire might be helpful gaining insight into the perceptions of *all* employees about many MCDs within an organization, for instance combined with observations of MCD sessions.

### Strengths and limitations

One of the strengths of this study was the fact that our observations and interviews were related to the same MCD sessions. Moreover, the interviewees were all directly involved in the case and close to practice. As such, the interviews were (also) used as a member check of the observations. Another strength of the study is that we were able to triangulate our data, as we used various forms of data collection: observations, memos, interviews and focus groups. A constant comparison among these various data sources was conducted, both with regard to the same MCD (within cases) and between MCDs (between cases). Another strength was our focus on the follow-up in practice, which is a relatively new approach to this research area. Next, a multiple-case study could take both the inherent value and as the utility of MCD into account, as was also mentioned previously [11, 13].

The main limitation of our study is that we were not able to interview clients or their relatives, as was initially planned. A first reason for this lack was that it turned out that only very few clients or relatives actually participated in or were aware of the MCD sessions conducted during our study period, although this participation was considered standard practice by the MCD working group in the organization. In some cases, the goal of the MCD was team-oriented, for instance, to reach consensus as a team concerning how to approach (relatives of) the client. Furthermore, in many observed MCDs, the moral issue referred to difficulties with the clients’ relatives, which raised the barrier to involving or inviting these relatives even further, at least in the perception of health care professionals. As a consequence, the presented findings are based on the perceptions of health care professionals. We do not yet know whether and how clients and their relatives took note of any impact of the MCD, or whether clients’ relatives recognized any impact on collaboration with them, for instance. Especially when discussing quality of care and quality of life, we lack the awareness of the views of clients and their relatives and need to be cautious in our concluding statements.

In addition, we did not participate in or observe the *actual care practice* before and after the observed MCDs, which could have deepened and complemented the perceptions of our interviewees. We recommend participatory observations for future studies on understanding the intersubjective impact of MCD. Relatedly, we did not set a clear timeframe for the period after which we interviewed the participants concerning their experiences and views on impact, as some were interviewed within two months and some at a later time after the MCD had taken place. We were constricted to the availability of interviewees and the limited duration of our project, but we acknowledge that our study does not show how impact might be dependent on the moment at which it is assessed.

Another limitation of this study is the fact that all the MCDs in this study took place on a single, one-off basis, while it has been suggested that MCD might only produce impact when it is frequently organized and that individual learning requires time [4]. Many participants in our study mentioned that it was the first MCD that they had experienced. This fact might have led to either an overestimation of the impact of MCD as they were simply enthusiastic about trying something new or an underestimation of that impact as they had not yet been able to encounter any impact resulting from their experience with MCD, since their moral learning needs to grow first. Nevertheless, our study shows that a *single MCD* can also have an impact on its participants and bring about practical consequences for the particular case under discussion.

### Implications for further research

Our findings indicate new grounds for further research into the impact of MCD. First, what counts as impact *(partly) caused by MCD* is not always clear and requires qualitative research methodology for the correct interpretation of what happens after the MCD session. Second, impact in general or high levels of impact in particular is not always normatively positive: no impact at all (i.e., the status quo) can also be considered to be a normatively positive impact. Third, what happens before the MCD (e.g., expectations towards and the assumed goals of the MCD session by its participants) already greatly influences the possibility and direction of the impact of MCD. Fourth, the impact and valuation of this impact are multi-interpretable: it is important to take into account the viewpoints of different stakeholders. It is therefore important to look at the preparation, content and process of MCD to assess and optimize its impact: to identify what elements are necessary for a good preparation for MCD and how this preparation is actually processed in terms of the content and conclusions of the MCD, for instance. As such, our study serves as a guideline for new MCD impact research by indicating new potential research topics, such as the ways in which the conclusions of MCD are translated into actual acts in practice and in the context of organizational policies as well as the contribution of MCD to fostering collaboration within the complete caring network, including client relatives. Our study thereby contributes to the further professionalization, efficacy and implementation of MCD – and ethics support services in general – in care practices.

## Conclusions

In our study, the impact of singular MCD sessions in the context of a long-term care facility was located in moral awareness and collaboration among participating health care professionals and between those professionals and clients’ relatives. Elements *before and during* MCD play a large role in establishing potential impact. Indicating impact in the actual client situation is complicated due to the many aspects that influence the MCD itself, the task of putting the conclusions of MCD into the practice, or the intentions, actions or events that occur between the MCD and actual practice. Hence, a conscious translational step might be needed between the goals and decisions made during MCD on the one hand and actual practical actions to better link the content, process and conclusions of the sessions to concrete follow-up in practice. In addition, the impact might be negative when misunderstandings arise among (vulnerable client) participants or when disappointments arise concerning the (lack of) follow-up. It is therefore important to consider ‘impact’ in a broad sense, including not only positive changes but also potential negative consequences or the possibility that nothing changes at all, in relation to the goals and context of the particular MCD in question. More insights are needed from the perspectives of clients and their relatives to enrich our findings regarding the impact on their relationships with health care professionals and on clients’ quality of care and life. Future studies should continue studying the link between the preparations, content and process of MCD on the one hand and the subsequent actual follow-up on these elements in practice, including the perspectives of clients and their relatives. For this purpose, a mixed-method approach is very suitable to investigate actual practice, which is the context in which the impact should take place and be noticeable.

## Supplementary Information

**Additional file 1:** Guide for observing the MCD sessions.

**Additional file 2:** Interview guide.

**Additional file 3:** Programme Focus group meeting.

## Data Availability

Data used and analysed during the current study (interview transcripts, observational notes and focus group summary) are available from the corresponding author on reasonable request.

## Abbreviation

MCD: Moral case deliberation
