# Supplementary material for: Conceptualizing the impact of moral case deliberation: a multiple-case study in a health care institution for people with intellectual disabilities
Source: BMC Med Ethics. 2022 Feb 5;23:10. doi: 10.1186/s12910-022-00747-2 (PMC8817498; doi:10.1186/s12910-022-00747-2)
Supplement: Supplementary file 1 — Additional file 1: Guide for observing the MCD sessions. [file 12910_2022_747_MOESM1_ESM.docx]

**Appendix I Guide for observing MCD sessions**

Content:

| **Work title of MCD/representative term** |  |
| --- | --- |
| **Part 1 Global overview of the MCD/summary** |  |
| **Part 2 Prior to the MCD (e-mail/phone contact)** |  |
| **Part 3 The MCD itself** |  |
| - A Space and objects - B Participants (actors) and their participation - C Describing and clarifying the case (activities and events) - D Considerations: perspectives and alternatives - E Balancing conclusions and decisions (goals) - F Atmosphere, emotions and feelings - G Role of facilitator |  |
| **Part 4 – Contact (shortly) after the MCD** |  |

Every part contained detailed questions to guide the observations. These questions were in Dutch and therefore not included in the overview presented here, but can be requested from the authors.
