# Supplementary material for: Conceptualizing the impact of moral case deliberation: a multiple-case study in a health care institution for people with intellectual disabilities
Source: BMC Med Ethics. 2022 Feb 5;23:10. doi: 10.1186/s12910-022-00747-2 (PMC8817498; doi:10.1186/s12910-022-00747-2)
Supplement: Supplementary file 2 — Additional file 2: Interview guide. [file 12910_2022_747_MOESM2_ESM.docx]

**Appendix II Interview Guide**

Interviews with care professionals after an MCD has taken place

Part I Introduction and informed consent

- Welcome and introducing each other
- Aim and structure of interview
- Confidentiality
- Informed consent form

Part II Looking back at the MCD session

1. Can you tell me something about the reason for the MCD? Why and by whom was it initiated?
2. How would you describe the situation of the client at that time?
3. Were you familiar with MCD or was it the first time?
4. Do you remember your expectations from the MCD?
5. What was your main concern? What did you worry about?
6. Can you shortly summarize how the MCD went? How was the atmosphere according to you?
7. How do you look back on the MCD? (including elements that were remarkable in the observation, if any)
8. What was the core of the MCD for you? (referring to the specific dilemma, concerns and values)
9. Do you remember your intuitive judgments and final opinion? Did it change, if so how?
10. Can you tell something about the conclusion or outcome of the MCD? Did you reach consensus, or plans for action?
11. How did you leave the MCD, what feelings/insights?
12. What did the MCD bring you, new insights? Was it useful?

Part III The period beyond the MCD up to now

1. Can you tell me something about the situation of the client, now and in the past period?
2. Did something change in caring for this client? If so, what and how? Can you give an example?
3. What has been done with the decisions made in the MCD (if any)?
4. Did something change in the collaboration with colleagues in the last period? If so, what and how? Can you give an example?
5. Did anything else change, for instance in collaboration with other stakeholders like family?
6. What do you find important in caring for this client? Why? And your colleagues?
7. Did you recall the MCD in the past period, for instance in a team meeting or so? How? What do people recall?
8. What is the difference between an MCD and a regular meeting?
9. How would it be if the MCD had NOT taken place?
10. What are your worries about the situation now? Did these change after the MCD? If so, how?
11. How did you report the MCD to the client or relatives?

Part IV Impact of MCD in general

1. How do you value MCD? Would you like to attend more in future?
2. What is the usefulness of MCD (if any)? Did it bring you something?
3. Can MCD have negative consequences, if so, what?
4. What are crucial preconditions for MCD according to you?
5. What should MCD lead to in this organization? What is needed for this, and what obstacles do you see?
6. How do you think about involving family/relatives in the MCD session? Would it have been possible for this particular case? Why (not)?

Closure

1. How was it to be interviewed?
2. Do you have any questions for us?
3. Do you have any suggestions for our research? What should we take into account?

Farewells plus invitation for further contact if needed.
