# Supplementary material for: Conceptualizing the impact of moral case deliberation: a multiple-case study in a health care institution for people with intellectual disabilities
Source: BMC Med Ethics. 2022 Feb 5;23:10. doi: 10.1186/s12910-022-00747-2 (PMC8817498; doi:10.1186/s12910-022-00747-2)
Supplement: Supplementary file 3 — Additional file 3: Programme Focus group meeting. [file 12910_2022_747_MOESM3_ESM.docx]

**Programme Focus group meeting – Impact of MCD**

Participants: facilitators of MCD

Moderator: JLPvG

Assistant: JdST

Duration: 2 hours

Prerequisites: written informed consent forms from all participants prior to start of audio-recording

**Program**

00.00-00.10 Welcome and introduction: short round to get to know each other.
Checking informed consent of all participants.
Goals of this session: to collect their views and experiences on impact of MCD

00.10-00.25 Presentation of (most prominent) findings from interviews and observations

00.30-01.30 Group interview part A: Collecting responses and additions to findings

Mind: safety: not judging or justifying findings but collecting input

- First responses: what do you think of this?
- Do you recognize it? Are you surprised?
  - What is remarkable?
  - What did you expect?
- Do the findings fit to your experiences?
  - What would you like to add to these findings?
    - Do you have an additional experience in which the contribution to quality of care was disappointing? If so, how did that happen?
    - Do you have an additional experience in which the contribution to quality of care was positive? How was that?
    - How could the impact of MCD be improved? What is needed for this?
  - What do you find difficult? How do you deal with that?

01.30-01.50 Group interview part B: the role of the facilitator and the role of MCD

- How do you see your own role as facilitator around involving client and family, and impact on quality of life and quality of care?
  - Now and in the ideal situation?
  - How is it to involve family/clients in MCD? What is difficult, and what is (extra) valuable?
  - What is the role of the facilitator in taking care that an MCD leads to a concrete decision? And regarding the follow-up? Or is this the responsibility of participants?
  - What needs do you have regarding this?
- How do you see the role of MCD in the care system for the client?
  - MCD often is something incidental within a long-term trajectory, how could we tackle its exact impact?
  - Do you think that a situation should improve after MCD? If so, how? What if this does not happen?

01.50-02.00 Summary and next steps
